# Germline cysts and asymmetry in early previtellogenic ovarian follicles in cultured albino females of sterlet *Acipenser ruthenus* L. 1758 (Chondrostei, Acipenseriformes)

**DOI:** 10.1007/s00709-019-01376-0

**Published:** 2019-04-24

**Authors:** Monika Żelazowska, Dorota Fopp-Bayat

**Affiliations:** 1 Department of Developmental Biology and Morphology of Invertebrates, Institute of Zoology and Biomedical Research, Jagiellonian University in Kraków, Gronostajowa 9, 30-387 Kraków, Poland; 2 Department of Ichthyology, Faculty of Environmental Science, University of Warmia and Mazury in Olsztyn, Oczapowskiego 5, 10-917 Olsztyn, Poland

**Keywords:** Oogonium (cystoblast), Germline cyst, Granular (Balbiani) cytoplasm, Follicular cells, Previtellogenesis

## Abstract

It is a first report on the structure of germline cells in ovaries of albino sterlet *Acipenser ruthenus* L. 1758. Ovarian nests, follicles, and germinal epithelium have been examined in gynogenetic and control specimens of this species. The structure of oogonia (named the cystoblasts) and of germline cysts in the nests has been described in detail. Also, the asymmetry in the cytoplasm and early growth of cystocytes in the cysts and of early previtellogenic oocytes has been described. In the cytoplasm of cystoblasts and in all cystocytes, a precursor of granular cytoplasm (Balbiani cytoplasm) is present and defines future vegetal region in the oocytes. Interestingly, the nuclei in cystoblasts comprise a large dense body that contains deoxyribonucleic acid (DNA). The role of this body in formation of multiple nucleoli has been explained. During the zygotene and pachytene stages, massive extrachromosomal amplification of DNA begins in the nucleoplasm of all cystocytes. As a result of the accumulation of extra DNA, an irregularly shaped DNA-body is formed. Multiple nucleoli arise in this DNA-body and around fragments of dense bodies. The asymmetry of the early previtellogenic oocyte cytoplasm is well marked by the presence of the granular cytoplasm. Moreover, the cisternae of the rough endoplasmic reticulum, dictyosomes, mitochondria, complexes of mitochondria with cement, nuage accumulations, and lipid droplets are located in specific zones in the granular cytoplasm. The follicular epithelium is composed of two subpopulations of somatic follicular cells (FCs): the main body cells and future micropylar cells.

## Introduction

Wild specimens of sterlet *Acipenser ruthenus* L. 1758 are sedentary and live in Eurasia, in the middle or lower parts in rivers (Billard and Lecointre 2001). Two races or subspecies of *A. ruthenus* have been distinguished: the European and Siberian ones (*A. ruthenus ruthenus* natio *marsigli* Brandt) (Kolman 2005). *A. ruthenus* comprises specimens that are the smallest among other species of sturgeons (family Acipenseridae). They grow slower but mature sexually faster (at age of 5–7 years) than other Acipenseridae and the lifespan of individuals of this species is the shortest. *A. ruthenus* is considered to be a diploid species and comprises ~ 60 macrochromosomes and 60 microchromosomes (Raikova 1976; Billard and Lecointre 2001; Fopp-Bayat and Woźnicki 2007). Recently, a study on the sex determination system in this species has revealed the female heterogametic sex (Fopp-Bayat et al. 2018). Although wild and pigmented specimens prefer bottoms of rivers with a very weak current, they lay eggs on the rocky and sandy bottom in strong current freshwater and also in flooded areas (Billard and Lecointre 2001; Kolman 2005). An interval between two successive spawnings in females lasts 1–3 years in the natural environment and 1–2 years in aquaculture (Raikova 1976; Billard and Lecointre 2001; Williot et al. 2005). Reproductive characteristics of Acipenseridae, including *A. ruthenus*, are very interesting and differ from those in Teleostei. They easily hybridize (in the natural environment and in aquaculture) and can produce viable offspring with different ploidy (Fopp-Bayat and Woznicki 2006; Fopp-Bayat et al. 2007;, 2017; Havelka et al. 2016). Moreover, the interspecific and intergeneric hybrids are fertile (Havelka et al. 2016). Another phenomenon identified in *A. ruthenus* is the occurrence of hermaphrodities that can be used for self-fertilization with a 0–70% survival of eggs (Williot et al. 2005).

Fish that suffer from albinism are unusual in the natural environment, from which they are quickly eliminated. They do not synthesize the black pigment, melanin, that defends against damage of genomes by UV irradiation in sunlight. Due to this failure, melanin is absent in the skin and in the iris. The lack of coloration in albinos causes increased sensitivity to environmental factors that may affect negatively their survival during the embryonic development and further stages of ontogenesis. Albino forms have been observed in Acipenseridae including *A. ruthenus* (Kolman et al. 2010; Fopp-Bayat and Ocalewicz 2015; Fopp-Bayat et al. 2017). Albino forms of *A. ruthenus* have also been artificially reproduced and reared in aquaculture. The eggs of albino sturgeons, including *A. ruthenus*, are used to produce the golden caviar (the most expensive food product). The ovulated eggs of albinos of this species are present and observed during artificial reproduction but structure of ovaries and of oocytes is not known.

The oocytes of fish (Pisces) grow within the ovarian follicles that arise in the germinal epithelium of the ovarian wall. Each follicle is composed of single oocyte that is surrounded by numerous somatic follicular cells. They derive from the somatic cells of the germinal epithelium and are covered by a basal lamina. Somatic thecal cells originate from somatic cells in the ovarian stroma and encompass the basal lamina (Selman and Wallace 1989; Le Menn et al. 2007). The ovaries of fish comprise also female germline stem cells present between somatic cells of the gonadal epithelium. The mitoses of germline stem cells are asymmetrical and lead to the formation of two progeny cells—a female germline stem cell and an oogonium (Selman and Wallace 1989; Nakamura et al. 2010, 2011; Elkouby et al. 2016; Elkouby 2017; Elkouby and Mullins 2017). Nuclei in the oogonia divide by mitoses but cytokineses are incomplete and the cytoplasms of progeny cells are not separated. The cysts composed of the progeny cells, named the cystocytes, are formed as a result of these divisions. The cystocytes remain connected by intercellular bridges that allow transport of molecules and organelles (Elkouby 2017; Elkouby and Mullins 2017). In the meroistic type of ovaries in vertebrates (i.e., in the house mouse *Mus musculus* and in the Siberian sturgeon *Acipenser baerii*) and also in invertebrates (insects and annelids), some cystocytes serve as the trophocytes (nurse cells) and “nourish” future oocytes. Their cytoplasm containing organelles and macromolecules (ribosomal RNA—rRNA, messenger RNAs—mRNAs, small nuclear RNAs—snRNAs and proteins) that are necessary during development of the embryo is transported to the cytoplasm (ooplasm) of differentiated oocyte. After the completion of this process, the trophocytes degenerate (Lei and Spradling 2016; Bilinski et al. 2017; Ikami et al. 2017; Urbisz et al. 2017; Żelazowska and Fopp-Bayat 2017b). In the panoistic type ovaries, all cystocytes differentiate into the oocytes (Bilinski et al. 2017).

Several papers report the presence of germline cysts in ovaries of Acipenseridae. However, the question whether the cytoplasm in cystocytes and in early previtellogenic (primary growth) oocytes in ovarian follicles is polarized and how the asymmetry develops remains not fully answered. Germline cysts are present in ovaries of Acipenseridae including *A. ruthenus*, Russian *A. gueldenstaedtii*, Siberian *A. baerii*, shortnose *A. brevirostrum*, and the Adriatic *A. naccarii* sturgeons (Raikova 1976; Raikova et al. 1979; Flynn and Benfey 2007; Grandi and Chicca 2008; Rzepkowska and Ostaszewska 2014; Żelazowska and Fopp-Bayat 2017b). In literature concerning the ovaries of vertebrates (i.e., fish and amphibians) and in invertebrates, the term cystoblast is frequently used and describes germline cell that is initially undifferentiated and that is potent to differentiate into female and male gametes (Nakamura et al. 2011; Bilinski et al. 2017). The cystoblast divides by incomplete mitoses which lead to formation of germline cysts (Kloc et al. 2004; Żelazowska et al. 2015; Bilinski et al. 2017; Tworzydlo et al. 2017). As the examined gonads of *A. ruthenus* have not been hermaphroditic and contained developing female germline cells (the oocytes), the term cystoblast refers here to the oogonium. In this paper, the cystoblasts in the ovarian nests and structure of cysts in albinos *A. ruthenus* have been comprehensively described. Also, the asymmetry in the cytoplasm and early growth of cystocytes and early previtellogenic ovarian follicles has been explained. This paper brings new data necessary during comparisons between Chondrostei and a model fish species, the zebrafish *Danio rerio* (Teleostei) that regard the development of germline cysts (Elkouby et al. 2016; Elkouby 2017; Elkouby and Mullins 2017). Cystoblasts in ovaries of the investigated *A. ruthenus* specimens represent the oogonia proliferate stage (mitosis) in Teleostei according to Selman and Wallace (1989) and Grier et al. (2018), and the cystocytes in germline cysts represent chromatin nucleolus stage. Previtellogenic oocytes in the ovarian follicles in the examined specimens represent the primary growth stage in Teleostei (multilple nucleoli, perinucleolar, and circumnucleolar oil droplets steps according to Grier et al. 2018).

## Material and methods

### Animals

Normal albino specimens of *A. ruthenus* and gynogenetic albino specimens of *A. ruthenus* were produced in Wasosze Fish Farm (Poland) by experimental reproducing of albino female and albino male (normal specimens) and by activation of eggs from albino female of *A. ruthenus* with UV-irradiated sperm collected from wild, colorated bester *Huso huso* and *Acipenser ruthenus* (gynogenetic specimens) according to the procedure described by Fopp-Bayat and Ocalewicz (2015). Experimental fish were reared in two separate tanks, in a RAS system, for 19 months post fertilization. The rearing procedure was described by Fopp-Bayat and Ocalewicz (2015), and Laczynska et al. (2017). After 19 months of rearing (in July 2016), the fragments of gonads from four normal albino and from four gynogenetic albino *A. ruthenus* were sampled for examination. The examined females were 17 months old and measured from 37 to 42 cm in length. They were anesthetized with 2-phenoxyethanol solution, euthanized by decapitation, and the gonads (ovaries) were dissected. The examined fragments of ovaries were taken from three isolated sections: cranially, medially, and caudally located.

### Light (LM) and transmission electron microscopy (TEM)

Fragments of ovaries were fixed in ice-cold 2.5% glutaric dialdehyde (Aldrich-Chemie) in 0.1 M phosphate buffer (pH 7.4). Following several days of fixation, they were rinsed and postfixed in 1% osmium tetroxide in 0.1 M phosphate buffer (pH 7.4) containing saccharose (5.6 g in 100 ml). Next, they were dehydrated in a series of ethanol and acetone and embedded in glycid ether 100 (Serva Electrophoresis). Semithin sections (0.7 μm) were stained with methylene blue in 1% borax and were photographed using a Leica DMR light microscope. Ultrathin sections (90 nm) were contrasted with uranyl acetate and lead citrate and were observed in transmission electron microscope (JEOL JEM 2100) at 80 kV.

### Histochemical analyses on semithin sections

Samples of ovaries were fixed in 4% formaldehyde. After fixation, they were rinsed, dehydrated in a graded series of ethanol, infiltrated, and embedded in histocryl acrylic resin (Agar Scientific). Semithin serial sections were stained in the dark with fluorescence dyes: diamidino-2-phenylindole dihydrochloride (DAPI, 3:100 for 45 min; Sigma-Aldrich) and with propidium iodide (1:800 for 60 min; Sigma-Aldrich). DAPI detects deoxyribonucleic acid (DNA). Propidium iodide detects ribonucleic acid (RNA) and DNA. Sections were photographed using a Leica DMR epifluorescence microscope (FLM) equipped with appropriate filters. The one-step procedure of silver impregnation described in Biliński and Bilińska (1996) to detect nucleolar organizers and Ag-NOR proteins was also applied. Images of silver stained semithin sections were enhanced with differential interference contrast (Nomarski’s contrast) and were taken under the same microscope.

## Results

**Fig. 1:**
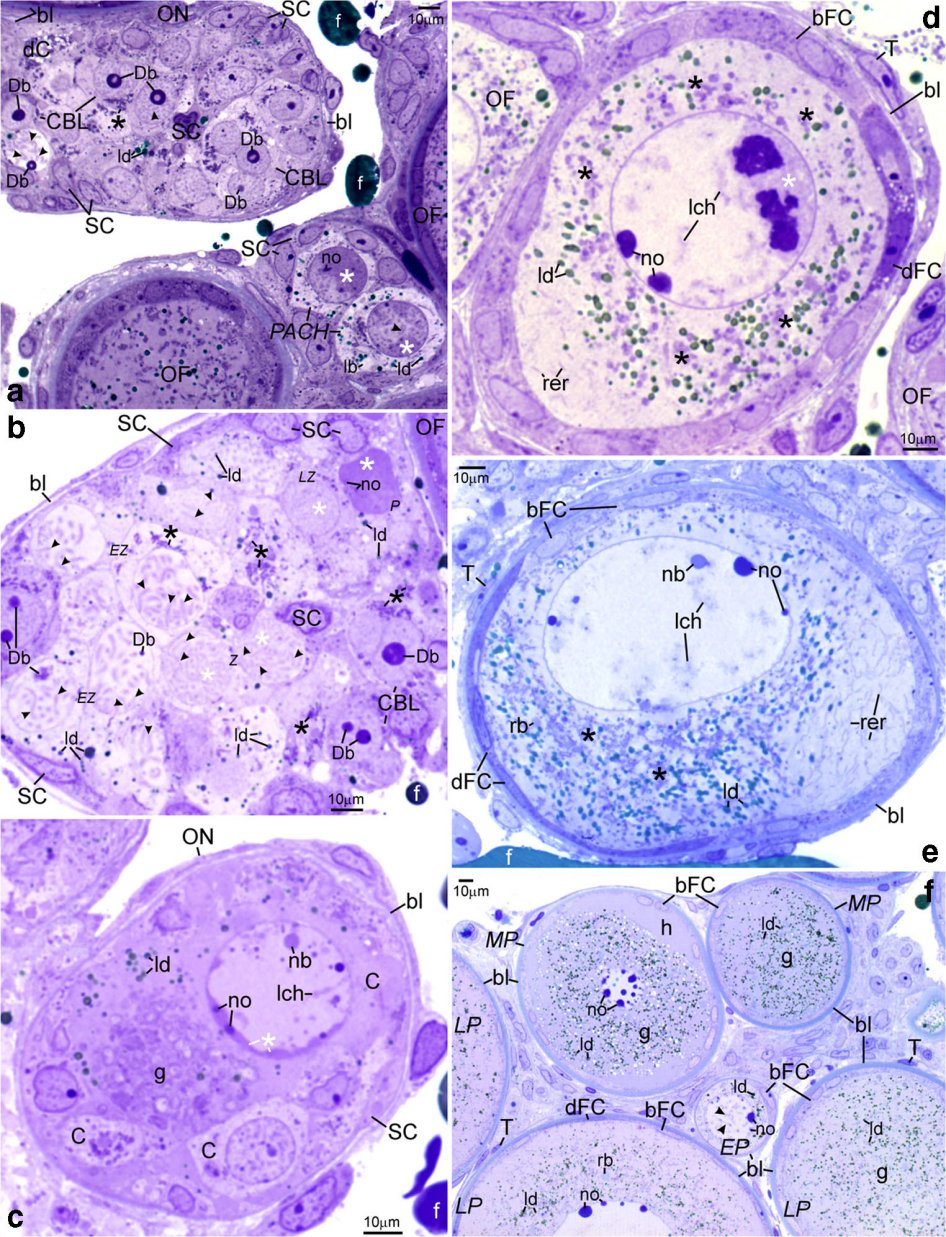
Albino *A. ruthenus*, semithin sections of ovary. *Abbreviations*: *ON*, ovarian nest; *OF*, ovarian follicle; *SC*, somatic cells; *bl*, basal lamina; *f*, fat tissue; *no*, multiple nucleoli; *ld*, lipid droplets; *rer*, rough endoplasmic reticulum; *bFC*, bright follicular cells; *dFC*, dark follicular cells; *T*, thecal cell. (**a**) Ovarian nests and follicles. *CBL*, cystoblast; *Db*, dense bodies; *arrowhead*, chromosome; *black asterisk*, precursor of granular cytoplasm; *dC*, degenerating cystocyte; *PACH*, pachytene stage oocytes; *white asterisk*, irregularly shaped DNA-body; *lb*, lucent body. (**b**) Female germline cyst in the ovarian nest. *CBL*, cystoblast; *EZ*, early zygotene stage cystocyte; *Z*, zygotene stage cystocyte; *LZ*, late zygotene stage cystocyte; *P*, pachytene stage cystocyte; *arrowhead*, bivalent; *Db*, dense body; *white asterisk*, extrachromosomal DNA in vicinity to bivalents; *black asterisk*, granular cytoplasm precursor. (**c**) Fragment of the cyst. *C*, cystocyte; *lch*, lampbrush chromosome in nucleoplasm of early diplotene stage cystocyte; *nb*, nuclear body; *white asterisk*, irregularly shaped DNA-body; *g*, granular cytoplasm. (**d** and **e**) *Black asterisks*, region in the granular cytoplasm that contains mitochondria, complexes of mitochondria with cement and nuage; *lch*, lampbrush chromosomes. (**d**) Early diplotene stage oocyte in early previtellogenic ovarian follicle; *white asterisk*, irregularly shaped DNA-body with embedded fused nucleoli. (**e**) Diplotene stage oocyte in early previtellogenic ovarian follicle; *nb*, nuclear body; *rb*, round body. (**f**) Early previtellogenic ovarian follicle (in the center, *EP*); *MP*, midprevitellogenic; *LP*, late previtellogenic follicles; *arrowhead*, lampbrush chromosome; *g*, granular cytoplasm; *h*, homogeneous cytoplasm. Methylene blue

**Fig. 2:**
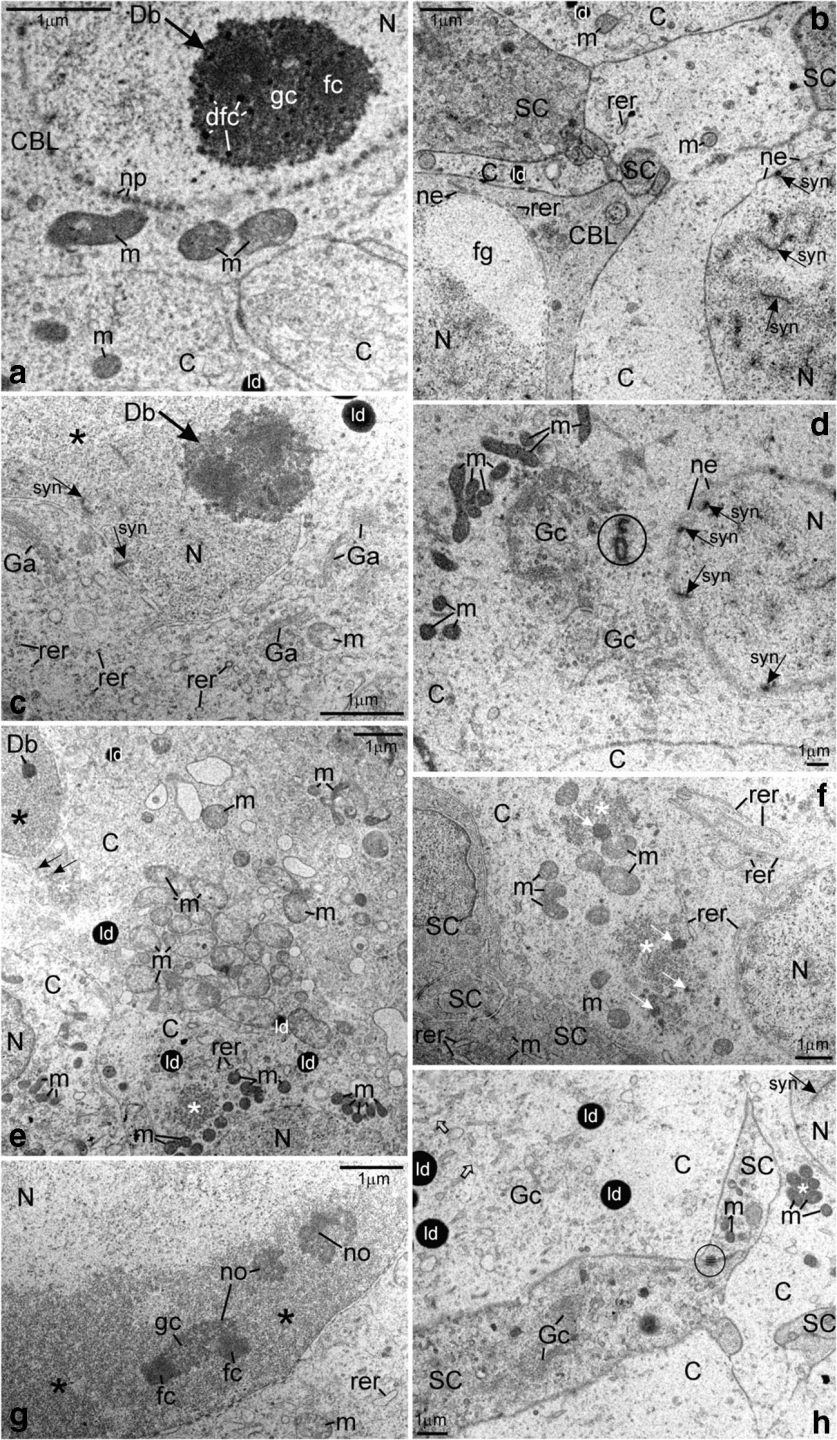
Albino *A. ruthenus*, ultrastructure of ovarian nests. *Abbreviations*: *C*, cystocyte; *N*, nucleus; *Db at arrow*, dense body; *syn at arrow*, synaptonemal complex; *ne*, nuclear envelope; *rer*, rough endoplasmic reticulum; *m*, mitochondria; *ld*, lipid droplet; *SC*, somatic cell. (**A**) *CBL*, cystoblast; *dfc*, dense fibrillar component of dense body; *fc*, fibrillar component of dense body; *gc*, granular component of dense body; *np*, nuclear pore. (**B**) *CBL*, cystoblast; *fg*, fibrillo-granular material; *C*, pachytene stage cystocyte. (**C**) Zygotene stage cystocyte. *Ga*, dictyosome; *asterisk*, extrachromosomal material in vicinity to bivalents. (**D**) Precursor of granular cytoplasm in the early zygotene stage cystocyte. *Gc*, Golgi complex; *encircled*, centrioles. (**E**) Two morphological types of mitochondria in the cytoplasm of zygotene stage cystocytes. *White asterisk*, fibrillar nuage in the cytoplasm; *arrow*, high electron-dense compact granules; *Db*, dense body; *black asterisk*, extrachromosomal material in vicinity to bivalents. (**F**) Early pachytene stage cystocyte at periphery of cyst. *Asterisk*, fibrillar nuage in the cytoplasm; *white arrow*, high electron-dense inclusion. (**G**) Pachytene stage cystocyte. *Asterisk*, irregularly shaped DNA-body; *no*, multiple nucleoli; *fc*, fibrillar component of nucleolus; *gc*, granular component of nucleolus. (**H**) Pachytene stage cystocytes and somatic cells. *White asterisk*, mitochondrial cement; *Gc*, vesicles and cisternae in Golgi complex; *empty arrow*, cytoskeleton; *encircled*, spot desmosome

**Fig. 3:**
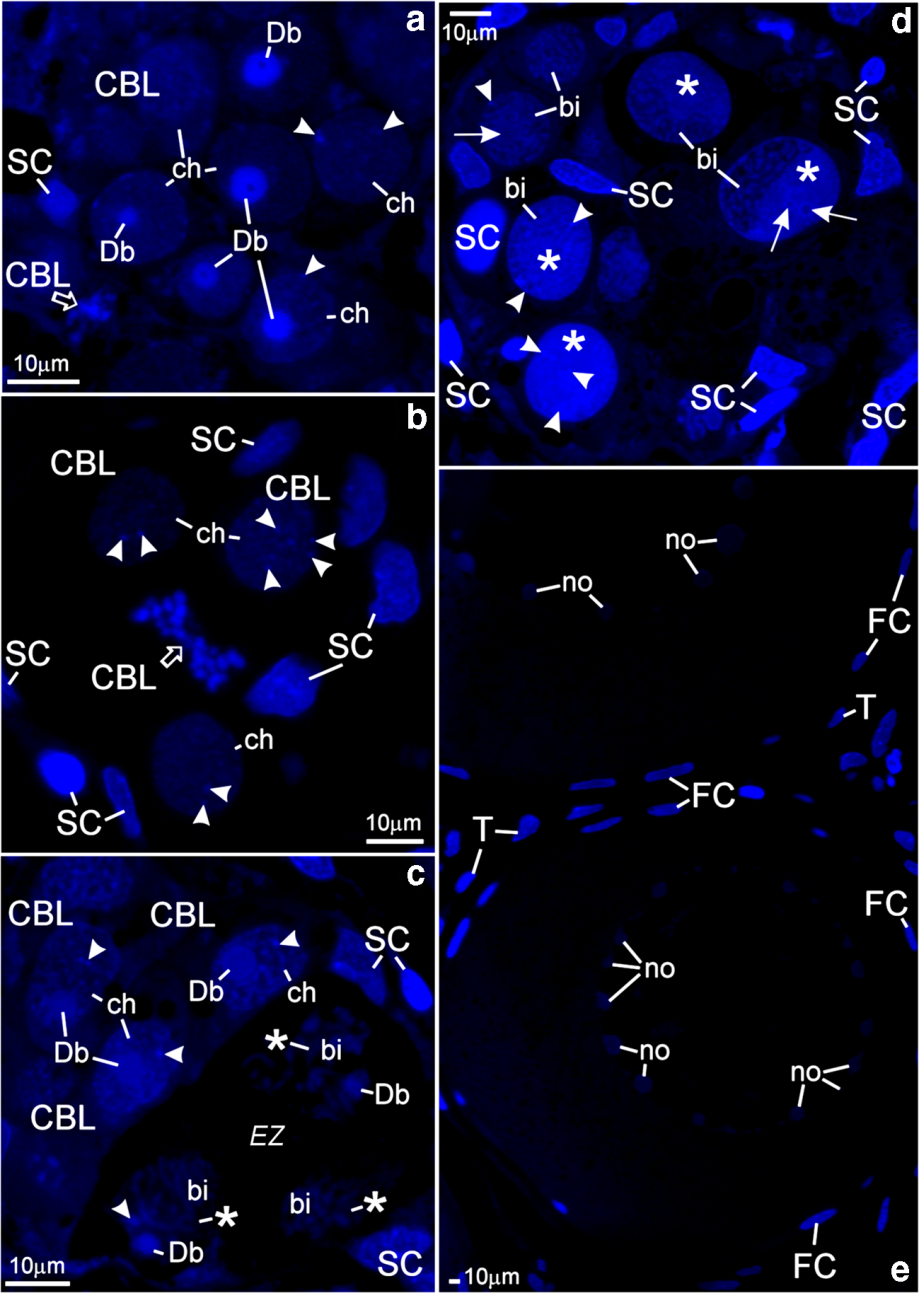
Albino *A. ruthenus*, DAPI staining of semithin sections of ovarian nests (**A**–**D**) and of individual previtellogenic ovarian follicles (**E**). *Abbreviations*: *Db*, dense bodies; *SC*, nuclei of somatic cells. (**A** and **B**) Nests containing cystoblasts; *CBL*, cystoblasts; *arrowheads*, spherical bodies; *ch*, chromosomes. (**A**) *Empty arrow*, chromosomes and fragments of DNA-body during mitotic division in the cystoblast. (**B**) *Empty arrow*, chromosomes and fragments of dense body during the anaphase in the cystoblast. (**C**) *CBL*, cystoblasts; *arrowhead* (at the cystoblast), spherical body; *ch*, chromosome; *EZ*, early zygotene stage cystocytes; *bi*, bivalents; *arrowhead* (at zygotene stage cystocyte), fragment of dense body; *asterisk*, extrachromosomal DNA. (**D**) Pachytene stage cystocytes. *Asterisk*, irregularly shaped DNA-body; *arrowhead*, fragments of dense body and of spherical bodies; *arrow*, multiple nucleolus; *bi*, bivalent. (**E**) Individual ovarian follicles; *no*, multiple nucleoli; *FC*, nuclei in follicular cells; *T*, nuclei in thecal cells

**Fig. 4:**
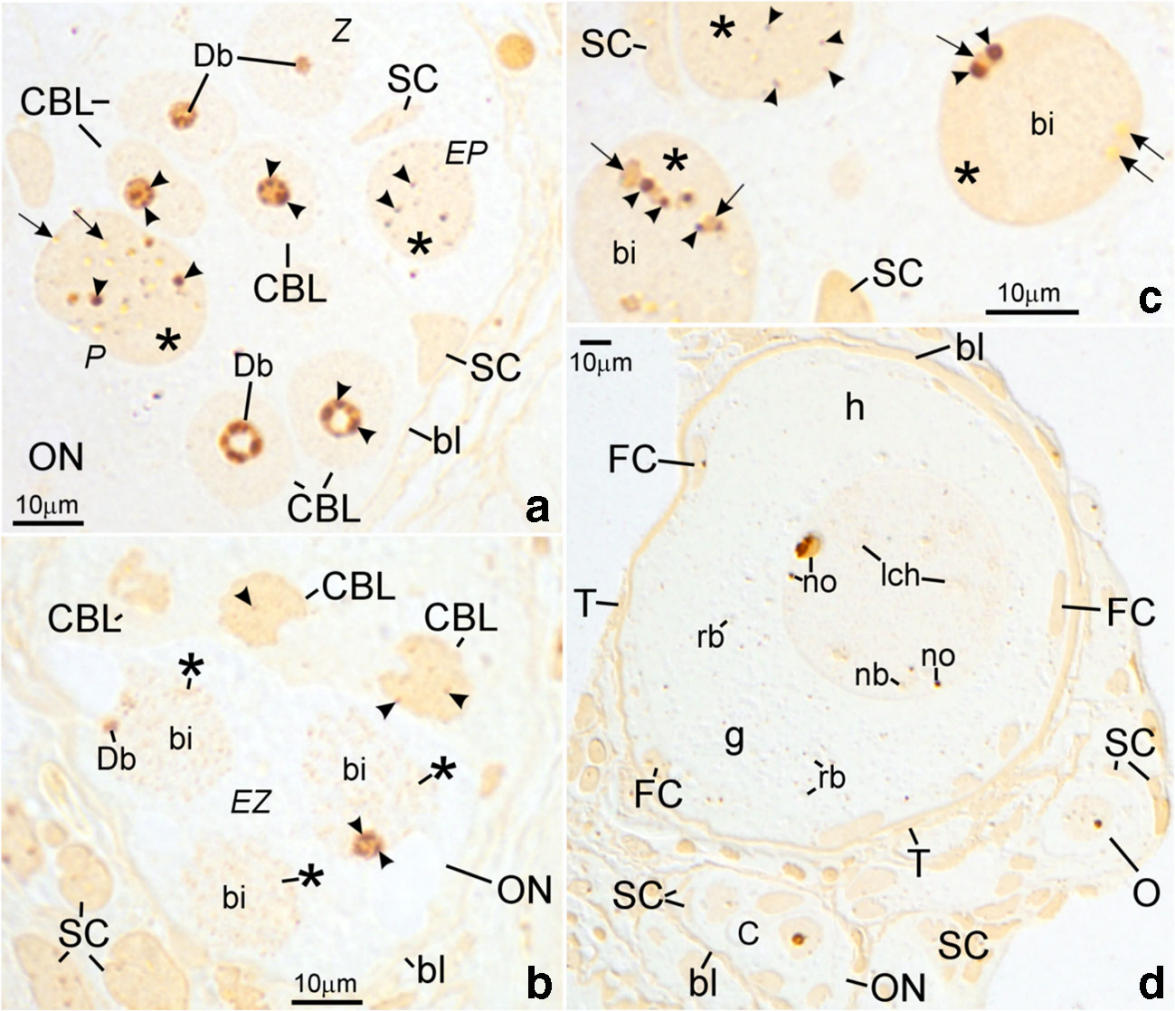
Albino *A. ruthenus*, silver impregnation in semithin sections of ovaries. *Abbreviations*: *bl*, basal lamina; *ON*, ovarian nest; *SC*, somatic cell. (**A**) *CBL*, cystoblast; *Z*, zygotene stage cystocyte; *EP*, early pachytene stage cystocyte; *P*, pachytene stage cystocyte; *Db*, dense bodies; *asterisk*, irregularly shaped DNA-body; *arrowhead* (at cystoblasts and at zygotene stage cystocytes), fibrillar component of dense body; *arrowhead* (at pachytene stage cystocytes), fibrillar component of the multiple nucleoli; *arrow*, granular component of multiple nucleoli. (**B**) *CBL*, cystoblast; *arrowhead* (at the cystoblast) spherical body; *EZ*, early zygotene stage cystocytes; *Db*, dense body; *arrowhead* (at zygotene stage cystocyte), fibrillar component of dense body; *bi*, bivalents; *asterisk*, extrachromosomal material. (**C**) Pachytene stage cystocytes. *Asterisk*, irregularly shaped DNA-body; *arrowhead*, fibrillar component of multiple nucleoli; *arrow*, granular component of multiple nucleoli; *bi*, bivalents. (**D**) Fragment of ovary. Individual early previtellogenic ovarian follicle contains diplotene stage oocyte; *T*, thecal cell; *FC*, follicular cell; *g*, granular cytoplasm; *h*, homogeneous cytoplasm; *rb*, round body; *no*, multiple nucleoli; *nb*, nuclear body; *lch*, lampbrush chromosomes; *C*, cystocyte; *O*, diplotene stage oocyte. Differential interference contrast (Nomarski’s contrast)

**Fig. 5:**
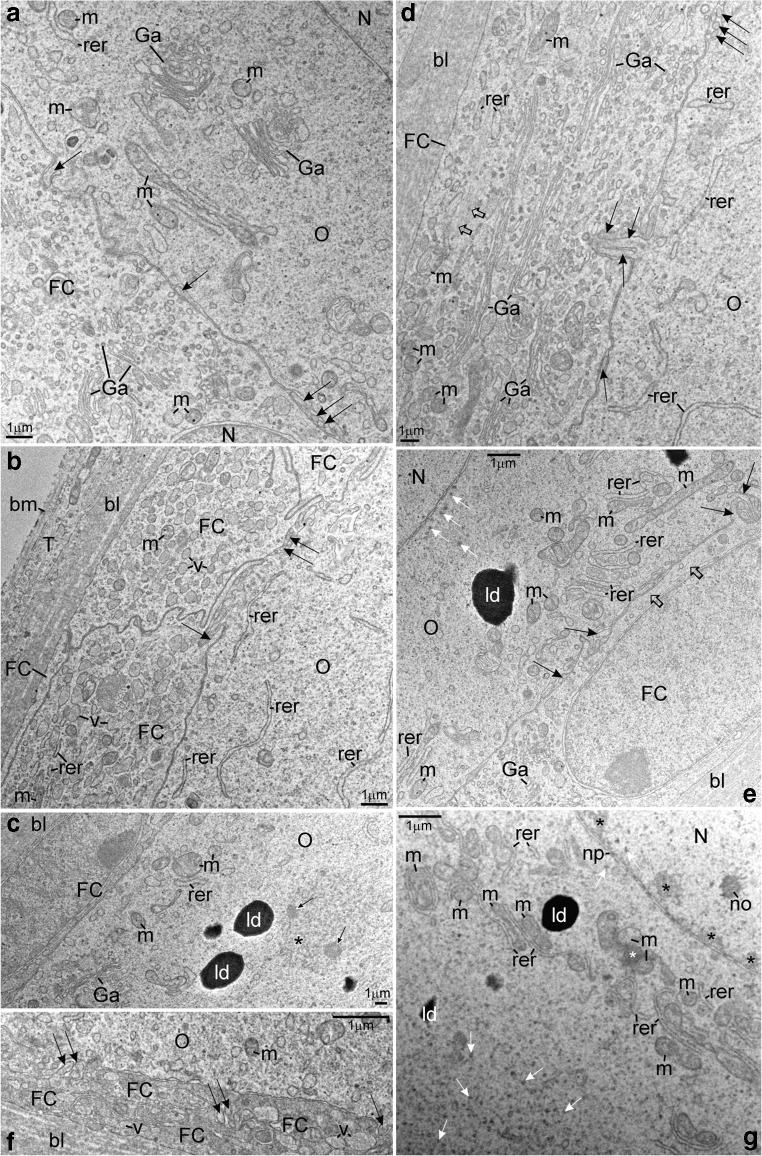
Albino *A. ruthenus*, ultrastructure of early previtellogenic ovarian follicles. *Abbreviations*: *O*, diplotene stage oocyte; *FC*, follicular cell; *bl*, basal lamina; *N*, nucleus; *Ga*, dictyosome; *rer*, rough endoplasmic reticulum; *m*, mitochondria; *ld*, lipid droplet. (**A**) Cytoplasm of bright FC and fragment of the granular cytoplasm in the oocyte. *Arrow*, microvillus. (**B**) Cisternae of rough endoplasmic reticulum in the region of ooplasm that is in vicinity to the oolemma. *T*, thecal cell; *bm*, basement membrane; *v*, Golgi vesicle; *arrow*, oocyte microvillus and process of bright FC. (**C**) *Asterisk*, fibrillar material in the round body; *arrows*, inclusions. (**D**) The bright FC. *Empty arrow*, cytoskeleton; *arrow*, microvillus. (**E**) *White arrows*, high electron-dense material of nuclear origin; *black arrow*, oocyte microvillus and process of bright FC; *empty arrow*, cytoskeleton. (**F**) Processes of lateral parts of dark FCs are intermingled; *v*, Golgi vesicle; *arrow*, microvillus. (**G**) Early diplotene stage previtellogenic oocyte in the ovarian follicle. *Black asterisks*, irregularly shaped DNA-body in contact with nuclear envelope; *no* multiple nucleolus; *white arrows*, high electron-dense material; *np*, nuclear pore; *white asterisk*, mitochondrial cement

**Fig. 6:**
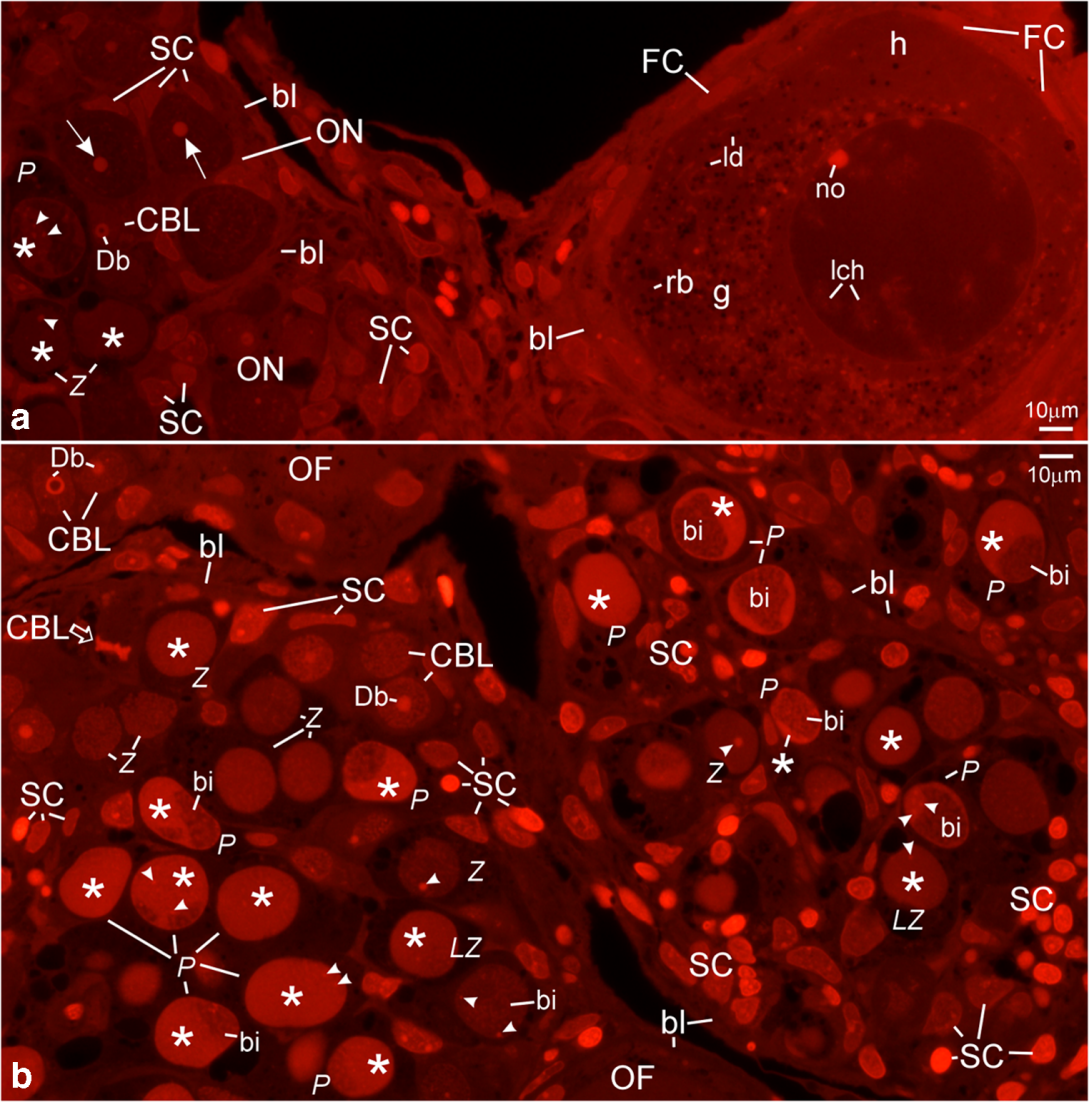
Albino *A. ruthenus*, propidium iodide staining of semithin sections of early previtellogenic individual ovarian follicle (**A**) and of ovarian nests (**A** and **B**). *Abbreviations*: *CBL*, cystoblasts; *Z*, zygotene stage cystocytes; *LZ*, late zygotene stage cystocytes; *P*, pachytene stage cystocytes; *Db*, dense bodies; *arrowhead* (at zygotene stage cystocytes), dense bodies; *arrowhead* (at pachytene stage cystocytes) multiple nucleolus; *asterisks*, irregularly shaped DNA-bodies; *SC*, somatic cells; *bl*, basal lamina. (**A**) *ON*, ovarian nest; *arrow*, nucleolus in the early diplotene stage oocyte; *FC*, follicular cells; *g*, granular cytoplasm; *h*, homogeneous cytoplasm; *ld*, lipid droplets; *rb*, round body; *no*, multiple nucleolus in diplotene stage oocyte; *lch*, lampbrush chromosomes. (**B**) *Empty arrow*, cystoblast at metaphase of mitosis; *bi*, bivalents; *OF*, neighboring ovarian follicle

**Fig. 7:**
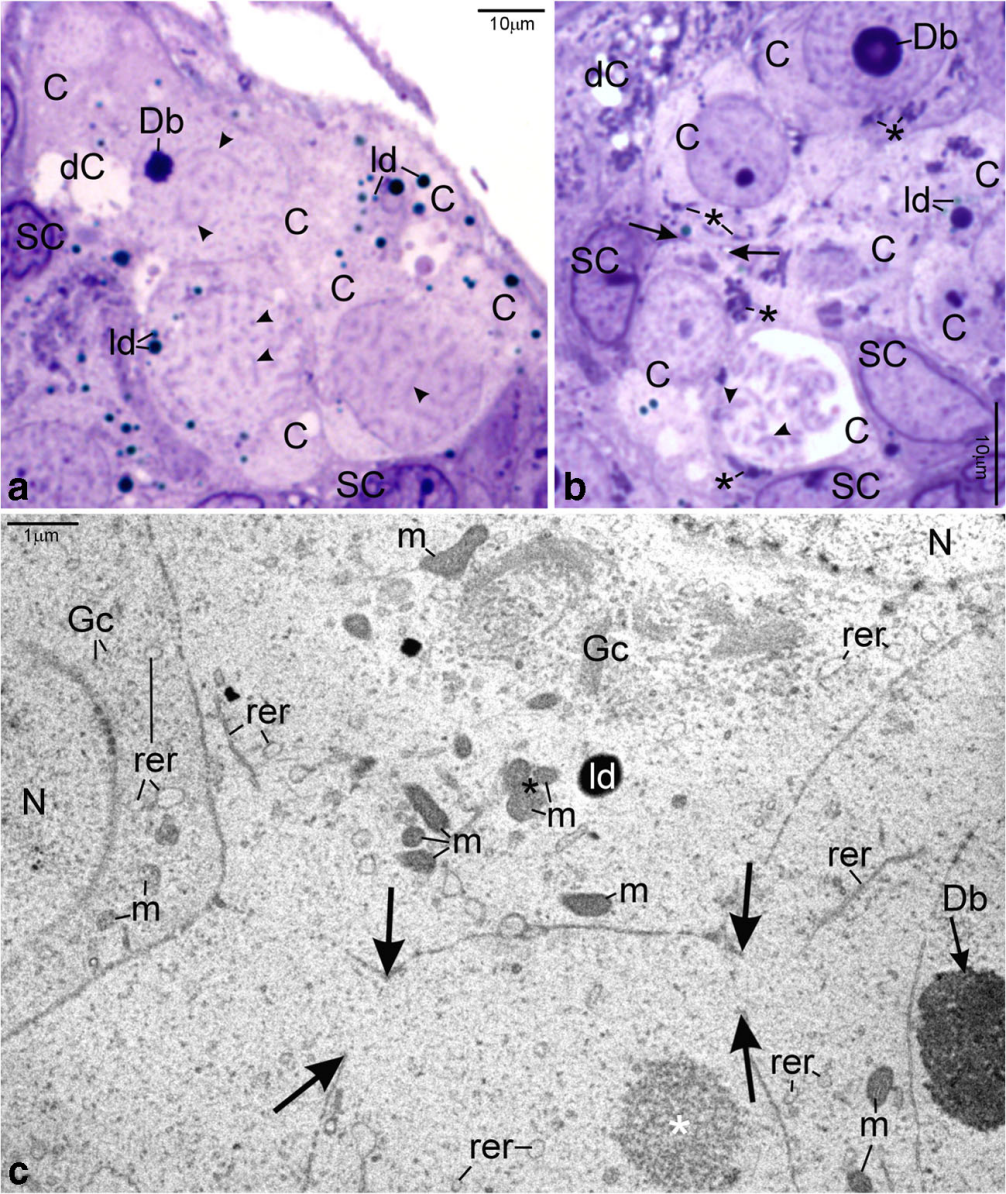
Albino *A. ruthenus*, intercellular bridges between cystocytes. *Abbreviations*: *C*, cystocyte; *Db*, dense body; *arrowhead*; bivalents; *ld*, lipid droplets; *dC*, degenerating cystocyte; *SC*; somatic cell. (**A**) Zygotene stage cyst. LM, methylene blue. (**B**) *Arrows*, rims of intercellular bridge; *asterisk*, granular cytoplasm precursor. LM, methylene blue. (**C**) *Arrows*, rims of intercellular bridges; *Gc*, Golgi complex; *m*, mitochondria; *rer*, rough endoplasmic reticulum; *ld*, lipid droplet; *black asterisk*, mitochondrial cement; *white asterisk*, fibrillar nuage; *N*, nucleus; *Db at arrow*, dense body. TEM

The ultrastructure of ovaries in albino gynogenetic and control specimens of *A. ruthenus* does not differ and the germline cells from these groups have been described together. Ovaries of all examined albinos contain the inner germinal epithelium and individual follicles (Figs. 1, 2, 3, 4, 5, 6, and 7). Ovarian nests have arisen in this epithelium. They comprise cystoblasts, cysts of female germline cells (cystocytes), and somatic cells (Figs. 1(A–C), 2, 3(A–D), 4, 6, and 7). Main bodies of most somatic cells are situated in the external walls of ovarian nests, while their processes are present between germline cells (Figs. 1(A–C), 2(B, F, H), and 7(A, B)). Each nest is covered by flat somatic cells, most probably future thecal cells (Fig. 1(A–C)). Female germline cysts are composed of 2 to 16 cystocytes depending on stage of cyst development. Their nuclei represent different stages of prophase I (leptotene, zygotene, and pachytene) (Figs. 1(B, C), 2, 3(A–D), 4(A–C), 6, and 7). In some nests, developing follicles that contain late pachytene and early diplotene oocytes are present. These oocytes are surrounded by flat somatic cells (Fig. 1(A, C)). Each individual ovarian follicle contains a previtellogenic oocyte in the diplotene stage that is surrounded by somatic follicular cells (FCs) and a basal lamina (Figs. 1(D–F), 3(E), 4(D), 5, and 6(A)). Flat thecal cells are present on this lamina (Figs. 1(D–F), 3(E), and 4(D)). Thecal cells are surrounded by extracellular matrix which is referred to as the basement membrane to distinguish it from the thick layer of extracellular matrix called basal lamina that surrounds somatic follicular cells (Fig. 5(B)).

### Cystoblasts

Cystoblasts are located between cells of the inner epithelium. They are single, in pairs, or sometimes appear as several cystoblasts neighboring each other. They are also present in ovarian nests, near their walls, and neighboring with female germline cysts (Fig. 1(A, B)). These cystoblasts are in direct contact with processes of somatic cells (Fig. 2(B)). The most striking feature of all cystoblasts is oval nuclei that comprise large, spherical, and centrally located dense bodies (Figs. 1(A, B) and 2(A)). Sometimes, these bodies are ring-shaped (Fig. 1(A, B)). Staining with DAPI is intense and indicates that dense bodies contain DNA (Fig. 3(A, C)). They impregnate with silver (Fig. 4(A)) and stain intensely with propidium iodide (Fig. 6(A, B)). The structure of dense bodies is almost identical to that of nucleoli. All dense bodies contain numerous, compact, and highly electron-dense fibrillar centers; a fibrillar component; and a granular component (Fig. 2(A)). Ag-NOR staining and TEM observation have revealed that fibrillar component in these bodies is also condensed and is embedded in granular component (Fig. 2(A) and arrowheads in Fig. 4(A)). Several smaller, spherical bodies that are DAPI-positive and impregnate strongly with silver are sometimes also present (arrowheads in Figs. 3(A–C) and 4(B)). The nucleoplasm in cystoblasts comprises chromosomes (arrowheads in Figs. 1(A, B) and 3(A–C)) and fine fibrillar material that contains granules. This material accumulates near the nuclear envelope (Fig. 2(B)). The cytoplasm comprises a precursor of the granular cytoplasm (Fig. 1(A), black asterisk). It consists of a few mitochondria, cisternae, and vesicles of the rough endoplasmic reticulum (RER) and Golgi apparatus (Fig. 2(A, B)). Two types of mitochondria have been distinguished: mitochondria with well-developed cristae and mitochondria with shortened and fused cristae (two types of mitochondria have been shown in zygotene stage cystocytes in Fig. 2(E)). The cytoplasm of cystoblasts does not impregnate with silver (Fig. 4(A, B)) and stains very weakly with propidium iodide (Fig. 6(A, B)).

Cystoblasts in the inner ovarian epithelium and those in the nests divide by mitoses (empty arrows in Figs. 3(A, B) and 6(B)). The mitoses lead to the formation of progeny cystoblasts. Nuclei in these cystoblasts continue divisions but cytokineses are not completed and the progeny cells (cystocytes) remain connected by intercellular bridges. Such divisions are continued at least four times and result in the formation of cysts (Figs. 1(B), 3(C), 4(B), 6(A, B), and 7(A–C)). During karyokineses, the dense bodies in cystoblasts split into fragments that are inherited by cystocytes and are incorporated into their nucleoplasm (empty arrows in Figs. 3(A, B) and 6(B)).

### Female germline cysts

Meiotic chromosomes in the nucleoplasm in cystocytes become attached to the nuclear envelope during the leptotene stage and the dense body is present in vicinity of the nuclear envelope until the late zygotene stage (Figs. 1(A, B), 2(C, E), 3(C), 4(A, B), 6(B), and 7(A–C)). During the zygotene, bivalents become connected by means of synaptonemal complexes (Fig. 2(C, D)) and medium electron-dense, loose, fibrillar material appears in their close vicinity (white asterisks in Fig. 1(B) and black asterisks in Fig. 2(C, E)). This material is DAPI-positive (contains DNA), impregnates weakly with silver (asterisks in Figs. 3(C) and 4(B)), and stains with propidium iodide (Fig. 6(A, B)). During the late zygotene, the entire nucleoplasm is filled with this material (Figs. 1(B) and 6(B), asterisks). At the beginning of pachytene stage, it condenses, acquires an irregular shape, and a DNA-body that is in contact with the nuclear envelope is formed (Figs. 1(B), 2(G), 3(D), 4(C), and 6(A, B), asterisks). Several multiple nucleoli arise in this DNA-body (Figs. 1(B) and 2(G); arrows in Fig. 3(D); and Figs. 4(C) and 6(A, B)). The fibrillar components of multiple nucleoli are usually separated and impregnate strongly with silver (Fig. 2(G) and arrowheads in Fig. 4(C)). They are connected by the common granular component that impregnates with silver less intensely (Fig. 2(G) and arrows in Fig. 4(C)). Sometimes, fibrillar components of nucleoli lie in their centers and are surrounded by the granular component (not shown). Although most of multiple nucleoli arise de novo during the pachytene, some are formed around fragments of dense bodies and spherical bodies that have been inherited. Some cystocytes enter the diplotene stage. Their nuclei contain irregularly shaped DNA-body, multiple nucleoli, nuclear bodies, and lampbrush chromosomes. At the vegetal region, the granular cytoplasm and several lipid droplets are present (Fig. 1(C)).

During the zygotene stage, all cystocytes are interconnected by strands of the common cytoplasm that are surrounded by plasma membrane (Fig. 7(A)). The cytoplasm comprises a few organelles, such as vesicles and cisternae of the RER, Golgi apparatus, mitochondria, lipid droplets, and medium electron-dense material (nuage) (Fig. 2(C, E)). The organelles in the cytoplasm in cystocytes form a precursor of the granular cytoplasm (Figs. 1(B) and 7(B), black asterisks). It comprises a pair of centrioles, which are in the vicinity of the nuclear envelope (Fig. 2(D), encircled). In their vicinity, Golgi complex is formed (Figs. 2(D) and 7(C)). In these cystocytes that are at peripheries of cysts, the centrioles are present close to cytoplasmic bridges (not shown). Cisternae and vesicles of the RER and mitochondria are also included in the precursor of granular cytoplasm (Figs. 2(D) and 7(C)). Cisternae of the RER are sometimes arranged parallel to both the nuclear envelope and plasma membrane and located in a very close vicinity of mitochondria (not shown). Mitochondria with fused cristae and mitochondria with well-developed cristae are present in the cytoplasm and in the granular cytoplasm precursor (Fig. 2(E)). Highly electron-dense lipid droplets appear in the cytoplasm in a very close vicinity of mitochondria that have fused cristae (Fig. 2(E)). Some mitochondria are in complexes with cement (Figs. 2(H) and 7(C), asterisks). Accumulations of medium electron-dense nuage are also present in the cytoplasm and in the precursor. Nuage is composed of loose, fine fibrillar material that comprises granules and highly electron-dense inclusions (Figs. 2(E, F) and 7(C), white asterisks). In many cases, the organelles in the precursor of the granular cytoplasm are present at this side of the nuclear envelope that neighbors with attached telomeres in the nucleoplasm (Figs. 2(C, D) and 7(B, C)). At peripheries of the granular cytoplasm and near the nuclear envelope, a lucent body that contains fine fibrillar material of nuclear origin is present (not shown). This material is DAPI-negative. Lucent bodies neither impregnate with silver nor stain with propidium iodide. The cytoplasm contains also microtubules and microfilaments (Fig. 2(H), empty arrows). Plasma membranes in neighboring cystocytes are separated by long processes of somatic cells (Fig. 2(B, F, H)). Neighboring somatic cells and their processes are connected by means of spot desmosomes (Fig. 2(H), encircled). Their cytoplasm contains the RER, dictyosomes, and mitochondria (Fig. 2(F, H)).

### Asymmetry in the granular cytoplasm in individual early previtellogenic ovarian follicles

Follicles that comprise early previtellogenic, diplotene stage oocytes are numerous in ovaries of both investigated albino groups (Figs. 1(D–F), 3(E), 4(D), 5, and 6(A)). Nuclei in the oocytes comprise lampbrush chromosomes, multiple nucleoli, and nuclear bodies (Figs. 1(D–F), 3(E), 4(D), and 6(A)). The DNA in loops of lampbrush chromosomes is loose and is not visible under a fluorescence microscope after staining with DAPI (Fig. 3(E)). These chromosomes impregnate with silver and stain with propidium iodide (Figs. 4(D) and 6(A)). All nuclear bodies are DAPI-negative, stain with propidium iodide, which indicates that they contain RNA (not shown), and impregnate with silver (Fig. 4(D)).

During the early diplotene stage, the irregularly shaped DNA-body that stays in contact with the nuclear envelope and comprises large and irregularly shaped nucleoli is present in the nucleoplasm. Smaller nucleoli are spherical and located in the vicinity of nuclear envelope (Figs. 1(D) and 5(G)). The RER cisternae in these oocytes are numerous and many of them are accumulated close to the oolemma (Fig. 1(D)). The cisternae that are close to centers of oocytes are associated with mitochondria (Fig. 5(G)). Some of these mitochondria form complexes with cement (white asterisk in Fig. 5(G)). In the vicinity of the nuclear envelope and in the granular cytoplasm, electron-dense granules of nuclear origin are present (Fig. 5(G), white arrows). During a more advanced diplotene stage, the oocyte nucleus is close to the oolemma (Figs. 1(E), 4(D), 5(A, E), and 6(A)). A few huge and spherical nucleoli arise in the nucleoplasm due to the fusion of smaller nucleoli (not shown). Sometimes, up to two or three small nucleoli are also present near the nuclear envelope (Figs. 1(E) and 6(A)). Their fibrillar components have irregular shapes, impregnate with silver intensely, and are surrounded by the granular component that impregnates with silver less intense (Fig. 4(D)). Organelles are located in specific regions in the granular cytoplasm (Figs. 1(E) and 5(A–F)). Many RER cisternae are arranged parallel and are close to the oolemma and neighbor with free ribosomes and a few mitochondria (Figs. 1(E) and 5(B, D)). Dictyosomes are present in the vicinity, in the granular cytoplasm (Fig. 5(A)). Some RER cisternae are present close to mitochondria and neighbor with the region in which the dictyosomes reside (Fig. 5(A)). Most of mitochondria and complexes of mitochondria and cement are in the center of the granular cytoplasm (Figs. 1(E) and 5(E)). These mitochondria are also close to the cisterneae of RER (Fig. 5(E)). Nuage accumulations (not shown) and round bodies are near the nuclear envelope, are DAPI-negative (Fig. 3(E)), and impregnate weakly with silver (Fig. 4(D)). The round bodies do not stain with propidium iodide, while nuage accumulations and ribosomes in the granular cytoplasm stain intensely (Fig. 6(A)). Round bodies are composed of compressed fine fibrillar, medium electron-dense material (asterisk in Fig. 5(C)) and contain electron-dense inclusions (arrows in Fig. 5(C)). In vicinity of the nuclear envelope, electron-dense granules are also present (white arrows in Fig. 5(E)). The homogeneous cytoplasm that comprises free ribosomes only is present in vicinity to the oolemma (Figs. 4(D) and 6(A)).

In the most voluminous early previtellogenic oocytes, the granular cytoplasm is close to the nucleus (at the vegetal side) and all organelles, nuage material, and round bodies are distributed randomly. In midprevitellogenic oocytes, the granular cytoplasm enlarges and surrounds the oocyte nucleus. The homogeneous cytoplasm is present close to the oolemma at its entire perimeter (Fig. 1(F)). In late previtellogenic oocytes, the granular cytoplasm and homogeneous cytoplasm blend together (Fig. 1(F)). The oolemma forms microvilli, which are grouped and present in the whole perimeter. Each group of microvilli is separated from the neighboring one by the smooth oolemma (Fig. 5(A, B, D–F)).

### Follicular cells in individual early previtellogenic ovarian follicles

The FCs surrounding the early diplotene stage oocytes and these in the more advanced diplotene are flat (Figs. 1(D–F) and 5(A–F)). Their nuclei are large and contain nucleoli and heterochromatin (Figs. 1(D–F) and 5(C, E)). Heterochromatin is DAPI-positive, impregnates silver, and stains with propidium iodide (Figs. 3(E), 4(D), and 6(A)). Characteristically, the FCs differ in their affinity to staining with methylene blue. The cytoplasm in the so-called dark FCs stains intensely; in the so-called bright FCs, the staining is less intense (Fig. 1(D–F)). In ovarian follicles that comprise advanced diplotene stage oocytes, these bright FCs are usually close to this region of the granular cytoplasm that contains the RER cisternae exclusively and may also cover the region in which dictyosomes are present (Fig. 1(E)). The dark FCs are usually close to the region in the granular cytoplasm that contains mitochondria that are associated with cisternae of RER and with cement (Fig. 1(E)). In mid- and in late previtellogenic ovarian follicles (comprise advanced diplotene stage oocytes), the shape of FCs does not change (Fig. 1(F)). The dark FCs are distributed randomly (Fig. 1(F)).

Two subpopulations of FCs are present: the main body cells and the future micropylar cells (Fig. 1(D–F)). The ultrastructure of these cells does not differ. The cytoplasm contains numerous cisternae and vesicles of the RER, dictyosomes, and vesicles that contain medium electron-dense material, mitochondria, ribosomes, and filaments (Fig. 5(A–F)). In apical parts of all FCs, i.e., in parts that are directed towards the oolemma, small concavities are present and the plasma membrane surrounds the oocyte microvilli (Fig. 5(A, D, F)). In these parts of FCs, also, a few microvilli-like processes are directed towards the oolemma (Fig. 5(B, E)). Lateral parts of FCs form elongated extensions that intermingle with each other and with oocytes’ microvilli (Fig. 5(B, F)).

## Discussion

Comparison of results obtained during this study with previous observations of ovaries in pigmented *A. ruthenus* specimens indicates that in albinos their ultrastructure does not differ (Raikova 1976; Raikova et al. 1979). However, the new data on the structure of *A. ruthenus* ovaries have been obtained during this study. They would allow better understanding of processes that take place during cysts formation and early previtellogenesis in sturgeons and paddlefishes (Acipenseriformes), the order of ancient and primitive fish (Bemis et al. 1997; Johnson et al. 2003; Zelazowska et al. 2007; Saito et al. 2014).

### Cystoblasts

Recently it has been shown that in *A. ruthenus* ovulated oocytes, the distribution of mRNAs *dnd*, *vasa*, and *ddx25* coding for germplasm determinants is uneven and shows increasing gradient towards the vegetal pole (Pocherniaieva et al. 2018). In this pole, the primordial germ cells (PGCs) develop (Ostaszewska and Dabrowski 2009; Saito et al. 2014). In Teleostei and also in Acipenseridae, during embryogenesis, the PGCs actively migrate, settle in primordia of gonads, and undergo mitotic divisions (Le Menn et al. 2007; Grandi and Chicca 2008; Ostaszewska and Dabrowski 2009; Lubzens et al. 2010; Mazzoni et al. 2010; Rzepkowska and Ostaszewska 2014; Saito et al. 2014; Grier et al. 2016). In developing ovaries of *A. gueldenstaedtii* and in *A. baerii*, mitotic divisions of PGCs result in the formation of two generations of cells: the early and the late PGCs. The oogonia arise due to mitotic divisions of late PGCs and continue mitoses. Some of these divisions lead to formation of germline cysts, the so-called nests of meiotic oocytes which are in the chromatin nucleus stage (leptotene to early diplotene oocytes) (Rzepkowska and Ostaszewska 2014). Two generations of PGCs (the so-called primary and secondary) are also present in putative ovaries of *A. naccari* (Grandi and Chicca 2008). Moreover, in this species, two generations of oogonia develop and are called the primary oogonia or oogonia-1 and the secondary oogonia or oogonia-2. The primary oogonia are single cells that are distributed between somatic cells in the germinal region of developing ovaries. The secondary oogonia start meiosis and form synchronous clusters of cells, the so-called early meiotic oocytes (Grandi and Chicca 2008). Two generations of oogonia develop also in ovaries of immature Gulf sturgeon, *A. oxyrinchus desotoi* (Grier et al. 2016). The so-called primary oogonia are small and occur between somatic cells in the germinal epithelium that lines lamellar surface. The secondary oogonia are larger and present within elongated sex cords. These cords are bounded by the basement membrane, which is continuous with the membrane that underlies cells in the germinal epithelium along the lamellar surface. Within these cords, prefollicular cells and early diplotene oocytes develop and form the ovarian follicles (Grier et al. 2016). The cystoblasts in examined *A. ruthenus* specimens are present both between somatic cells in the germinal epithelium near the external surface of ovaries and in the ovarian nests. These nests correspond to the sex cords in *A. oxyrinchus*.

In cystoblasts in the examined species, the early asymmetry has been determined. Such a situation has also been described in cystoblasts in ovaries of the African clawed frog *Xenopus laevis*. In this species, the cystoblasts, cystocytes, and oocytes comprise the Balbiani body, which is responsible for the formation of the germplasm at the vegetal region or the hemisphere (Kloc et al. 2004; Kloc and Etkin 2005; Bilinski et al. 2017). The Balbiani body is composed of the RER, Golgi complexes, mitochondria, mitochondria in complexes with cement, and nuage, and it carries localized maternal mRNAs (Kloc and Etkin 2005). The Balbiani body has also been described in the ooplasm in *Danio rerio*, Atlantic salmon *Salmo salar*, in mammalian *M. musculus*, and in numerous insect species (Kloc et al. 2001, 2008; Marlow and Mullins 2008; Elkouby et al. 2016; Lei and Spradling 2016; Škugor et al. 2016; Bilinski et al. 2017; Elkouby 2017; Elkouby and Mullins 2017). The cystoblasts in *A. ruthenus* comprise a precursor of the granular cytoplasm. The granular (Balbiani) cytoplasm in oocytes of Acipenseridae is considered homologous to the *X. laevis* Balbiani body (Zelazowska et al. 2007; Ye et al. 2015). The organelle components of this precursor and nuage are also present in cystoblasts (oogonia) in *A. gueldenstaedtii* and in the secondary oogonia in *A. naccarii* (Raikova 1976; Grandi and Chicca 2008). In *A. ruthenus*, the precursor comprises the mitochondria with shortened cristae. Such degenerating mitochondria are also present in previtellogenic oocytes in *A. gueldenstaedtii* and in the North American paddlefish *Polyodon spathula* (family Polyodontidae) and are eliminated. In contact with these mitochondria, in these zones where the granular cytoplasm resides, a lipid body composed of lipid droplets is formed (Żelazowska and Kilarski 2009; Żelazowska et al. 2015). The nucleoplasm in cystoblasts in *A. ruthenus* contains prominent dense body; at the opposite side of the nucleus, the fine fibrillar material accumulates near the nuclear envelope, from where it is subsequently transported to the cytoplasm and stored in lucent bodies. Lucent bodies are also present in the cytoplasm of cystocytes and oocytes in *A. gueldenstaedtii* and in *A. baerii*; their role is unknown (Raikova 1976; Żelazowska and Fopp-Bayat 2017b).

### Female germline cysts

During the leptotene and zygotene stages, asymmetry in the cytoplasm establishes in *A. ruthenus*. The components of the granular cytoplasm are assembled around the pair of centrioles that are near the outer nuclear membrane at vegetal region. In this assemblage, there are several dictyosomes located the closest to centrioles and form the Golgi complex. Similar localization of centrioles and of Golgi complexes has been observed in the Balbiani bodies in cystocytes in *M. musculus* (Kloc et al. 2008; Lei and Spradling 2016). Also, the centrioles in *X. laevis* and in *D. rerio* are involved in the arrangement of the Balbiani body in cystocytes (Kloc et al. 2004; Elkouby et al. 2016; Bilinski et al. 2017; Elkouby 2017; Elkouby and Mullins 2017). In panoistic ovaries of *X. laevis*, all cystocytes start meiosis and eventually differentiate into oocytes (Kloc et al. 2004; Bilinski et al. 2017). In the meroistic type ovaries, i.e., in *M. musculus*, the centrioles and dictyosomes are transferred through intercellular bridges to the cytoplasm of only one cystocyte in the cyst, to the one which differentiates into the oocyte (Lei and Spradling 2016; Ikami et al. 2017). Cytoplasm in intercellular bridges in *A. baerii* comprises vesicles and cisternae of the RER (Żelazowska and Fopp-Bayat 2017b). Intercellular bridges that connect cystocytes in the examined *A. ruthenus* ovaries allow transport of large organelles such as the mitochondria and dictyosomes. Moreover, in the cytoplasm of *A. ruthenus* cystocytes, during the pachytene stage, the dictyosomes and vesicles in Golgi complexes become less compact than in the previous stage. Next, they become dispersed in the cytoplasm, close to plasma membrane and to intercellular bridges. Although during this stage of development of *A. ruthenus* ovaries, the degenerating cystocytes are rare, it cannot be excluded that some cystocytes transfer their organelles (including dictyosomes) to the cytoplasm of future oocytes and subsequently die identically as do the trophocytes in meroistic ovaries. Numerous degenerating cystocytes have been present in ovaries of *A. naccarii* and in *A. baerii* (Grandi and Chicca 2008; Żelazowska and Fopp-Bayat 2017b). Moreover, in *A. baerii*, the DNA-bodies in the nucleoplasm in late pachytene cystocytes that are to degenerate undergo a characteristic transformation, i.e., they lose contact with nuclear envelope, and their cytoplasm contains numerous large lipid droplets almost exclusively. These degenerating cystocytes in *A. baerii* possibly had transferred most of their organelles and nuage to future oocytes through intercellular bridges (Żelazowska and Fopp-Bayat 2017b). Numerous large lipid droplets, mitochondria with abnormal cristae, multilamellar bodies, and the so-called cytoplasmic gaps have also been present in the ooplasm of degenerating meiotic oocytes of *A. naccarii* (Grandi and Chicca 2008). In this species, these meiotic oocytes correspond to and are the same as the cystocytes in ovaries of *A. ruthenus*. In all Acipenseridae and in Polyodontidae investigated so far, after the period of development within the cysts in ovarian nests, the oocytes grow in individual ovarian follicles without any support from other germline cells, and ovaries represent the panoistic type (Raikova 1976; Chen et al. 2006; Flynn and Benfey 2007; Zelazowska et al. 2007, 2015; Grandi and Chicca 2008; Żelazowska and Kilarski 2009; Rzepkowska and Ostaszewska 2014; Żelazowska and Fopp-Bayat 2017a, b).

### Dense bodies, DNA-body, and multiple nucleoli in nucleoplasm of cystocytes

Previous studies on the extrachromosomal amplification of ribosomal DNA (rDNA) in pachytene stage oocytes of Acipenseridae, in the late 1970s, were carried out on wild and adult specimens of *A. ruthenus* and *A. gueldenstaedtii* (Raikova et al. 1979). The amplification of rDNA leads to formation in the nucleoplasm of the so-called extra DNA that contains amplified and extrachromosomal rDNA cistrons (Coimbra and Azevedo 1984). The accumulations of amplified rDNA in the nucleoplasm are commonly referred to as the DNA-bodies (Coimbra and Azevedo 1984; Kubrakiewicz and Biliński 1995; Tworzydło and Biliński 2008; Żelazowska and Fopp-Bayat 2017b) or, less frequently, the caps (Raikova 1976; Raikova et al. 1979; Thiry and Poncin 2005). In *A. gueldenstaedtii*, the caps are initially formed and present in the vicinity of the so-called primary nucleoli (Raikova 1976). In cultured *A. baerii* specimens, the DNA-bodies are present in the nucleoplasm of pachytene stage cystocytes and their role in the formation of multiple nucleoli has been confirmed (Żelazowska and Fopp-Bayat 2017b). In *X. laevis*, the extrachromosomal amplification of rDNA genes also takes place, but the DNA-bodies remain undetectable in cystoblasts and in cystocytes until the pachytene stage because they are quickly enclosed in the multiple nucleoli (Coimbra and Azevedo 1984). In the nucleoplasm in oocytes of Teleostei, multiple nucleoli are universally present during the pachytene and diplotene stages (Selman and Wallace 1989; Thiry and Poncin 2005; Le Menn et al. 2007).

Cystoblasts that are present in the examined ovaries of *A. ruthenus* contain a spherical, solid, or ring-shaped dense body that is identical to the primary nucleolus according to Raikova (1976) in the nucleoplasm of wild *A. ruthenus* specimens. It contains DNA, the most probably extra DNA. In the examined albino specimens, the cap is not formed in the vicinity of the dense body. Instead, during four subsequent mitotic divisions of the cystoblast, the dense body becomes fragmented and fragments disperse in the nucleoplasm. They are inherited by all 16 progeny cystocytes. New ring-shaped dense bodies which also contain the DNA are formed around these fragments in the nucleoplasm during leptotene and zygotene stages in nucleoplasm of cystocytes. These dense bodies are present in the vicinity of chromosomes and close to the nuclear envelope. During the zygotene stage, the massive amplification of rDNA is initiated. Numerous new copies of rDNA genes are deposited in the nucleoplasm in the vicinity of bivalents, and eventually the irregularly shaped DNA-bodies are formed and start to enlarge in the nucleoplasm. Concomitantly, the ring-shaped dense bodies become fragmented. They contain fragments of dense bodies that have been inherited from the cystoblast and become incorporated and embedded in these enlarging irregularly shaped DNA-bodies. Multiple nucleoli arise in the irregularly shaped DNA-bodies and around fragments of dense bodies during the late pachytene and early diplotene stages.

### Asymmetry in individual early previtellogenic ovarian follicles

Oocytes that develop in individual ovarian follicles in ovaries of Acipenseridae are distinctly polarized (Raikova 1976; Le Menn and Pelissero 1991; Zelazowska et al. 2007; Żelazowska and Kilarski 2009; Siddique et al. 2014; Ye et al. 2012, 2015, 2018; Yue et al. 2014; Yang et al. 2015; Żelazowska and Fopp-Bayat 2017a, b). In early previtellogenic oocytes of *A. ruthenus*, the asymmetry is also evident. The granular cytoplasm is distinct and is transiently polarized. Initially (early diplotene stage), the RER and mitochondria that are in the vicinity of the nuclear envelope come into close contact. Next, many cisterneae of RER, mitochondria, and mitochondria that are in complexes with cement become relocated to the center of the granular cytoplasm. There, the RER cisternae contact with dictyosomes and cooperate. The rest of RER reside in vicinity to the oolemma.

The Golgi vesicles in *M. musculus* form in this region of the oocyte in which the cytoplasmic bridge connecting the cystocytes had previously formed (Kloc et al. 2008). Recently, it has been shown that in quiescent primary oocytes that belong to a pool of resting primordial follicles in ovaries of *M. musculus*, the Golgi complex is highly compact and located at the periphery of Balbiani body. It plays a role of a physical barrier that stops certain cytoplasmic components inside the Balbiani body for some time (Ikami et al. 2017). These arrested components comprise the mouse Vasa homologue (MVH), which is a component of the germplasm, and a Trailer hitch RNP, a conserved ribonucleoprotein, a member of the protein complex that is associated with the endoplasmic reticulum and Golgi vesicles and is responsible for the transport and metabolism of RNAs in the oocytes (Pepling et al. 2007; Ikami et al. 2017). In oocytes of *M. musculus* that reinitiate the development, the Golgi complexes acquire reticular structure and lose intimate connection with other organelles in the Balbiani body (Ikami et al. 2017). The granular (Balbiani) cytoplasm in Acipenseriformes expands towards the oocyte periphery where it blends in with the homogenous (organelle-free) cytoplasm (Zelazowska et al. 2007; Żelazowska and Kilarski 2009; Żelazowska and Fopp-Bayat 2017a). The dictyosomes in Golgi complexes in vicinity to the RER and to complexes of mitochondria with cement in granular cytoplasm in the early previtellogenic diplotene stage oocytes in ovarian follicles of *A. ruthenus*, *A. gueldenstaedtii*, *P. spathula*, and *A. baerii* may also play the same role as in the Balbiani body in oocytes of *M. musculus* (Zelazowska et al. 2007, 2015; Żelazowska and Fopp-Bayat 2017a). This role comprises the posttranslational modification of proteins synthetized on endoplasmic reticulum and, possibly, the temporal separation of some germplasm proteins and RNAs from the rest of the cytoplasm and delivery of certain components of the oolemma or extracellular matrix (including components of the eggshell) to specific regions of the ovarian follicle.

In *A. ruthenus*, multiple nucleoli in the nucleoplasm fuse and a huge nucleolus is formed during this phase. In early previtellogenic oocytes in individual ovarian follicles in *A. baerii*, the nucleoplasm comprises up to two or three huge nucleoli and the ooplasm contains numerous free ribosomes (Żelazowska and Fopp-Bayat 2017a, b). The nucleoplasm of early diplotene stage oocytes that initiate primary growth in *A. oxyrinchus* also comprises such huge nucleoli. In this species, the ribosomes accumulate close to the inner nuclear membrane, and next are transported to the ooplasm (Grier et al. 2016).

### Follicular cells

The FCs diversify during the oocyte growth in *A. gueldenstaedtii* and in *A. baerii* ovaries. In vitellogenic oocytes of these species, three subpopulations of FCs are distinguished: animal region cells, micropylar cells, and main body cells that cover vegetal region in the oocytes (Żelazowska 2010; Żelazowska and Fopp-Bayat 2017a). Micropylar cells are distributed between animal region cells, in the so-called micropylar field at the animal pole, and are equipped with long projections that are oriented towards the oolemma (Debus et al. 2002; Psenicka et al. 2010; Żelazowska 2010). In early previtellogenic ovarian follicles of *A. ruthenus*, the ultrastructure of micropylar cells does not differ from that in the main body cells and projections cannot be distinguished. In Acipenseriformes, the number of micropylar cells differs between species, may be different between specimens, and may also vary between eggs in one female. Consequently, the eggs are equipped with multiple micropyle consisting of numerous, mutually connected micropylar canals (Cherr and Clark 1982; Linhart and Kudo 1997; Debus et al. 2002; Siddique et al. 2014). A completely different situation occurs in ovaries of Teleostei in which only one micropylar cell is present and the eggshell contains single micropylar canal formed around its projection (Kunz 2004). In ovaries of *D. rerio*, a *bucky ball* gene (*buc*) is necessary for determination of this micropylar cell at the animal pole and for the formation of precursors of germinal granules in the ooplasm (Marlow and Mullins 2008). In Dabry’s sturgeon *A. dabryanus*, *buc* gene functions in germplasm formation (Ye et al. 2018).
